# Factors Influencing the Dosimetry for High-Intensity Focused Ultrasound Ablation of Uterine Fibroids: A Retrospective Study

**DOI:** 10.1097/MD.0000000000000650

**Published:** 2015-04-03

**Authors:** Song Peng, Lian Zhang, Liang Hu, Jinyun Chen, Jin Ju, Xi Wang, Rong Zhang, Zhibiao Wang, Wenzhi Chen

**Affiliations:** From the State Key Laboratory of Ultrasound Engineering in Medicine Co-founded by Chongqing and the Ministry of Science and Technology (SP, LZ, LH, JC, ZW), Chongqing Key Laboratory of Ultrasound in Medicine and Engineering, College of Biomedical Engineering, Chongqing Medical University; HIFU Center for Tumor Therapy (JJ, XW, RZ), 1st Affiliated Hospital of Chongqing Medical University; and Clinical Center for Tumor Therapy (WC), 2nd Affiliated Hospital of Chongqing Medical University, Chongqing, China.

## Abstract

The aim of this article is to analyze factors affecting sonication dose and build a dosimetry model of high-intensity focused ultrasound (HIFU) ablation for uterine fibroids.

Four hundred and three patients with symptomatic uterine fibroids who underwent HIFU were retrospectively analyzed. The energy efficiency factor (EEF) was set as dependent variable, and the factors possibly affecting sonication dose included age, body mass index, size of uterine fibroid, abdominal wall thickness, the distance from uterine fibroid dorsal side to sacrum, the distance from uterine fibroid ventral side to skin, location of uterus, location of uterine fibroids, type of uterine fibroids, abdominal wall scar, signal intensity on T2-weighted imaging (T2WI), and enhancement type on T1-weighted imaging (T1WI) were set as predictors to build a multiple regression model.

The size of uterine fibroid, distance from fibroid ventral side to skin, location of uterus, location of uterine fibroids, type of uterine fibroids, signal intensity on T2WI, and enhancement type on T1WI had a linear correlation with EEF. The distance from fibroid ventral side to skin, enhancement type on T1WI, size of uterine fibroid, and signal intensity on T2WI were eventually incorporated into the dosimetry model.

The distance from fibroid ventral side to skin, enhancement type on T1WI, size of uterine fibroid, and signal intensity on T2WI can be used as dosimetric predictors for HIFU for uterine fibroids.

## INTRODUCTION

As a noninvasive modality for uterine fibroids, high-intensity focused ultrasound (HIFU) ablation has been applied in an increasingly broader clinical setting because of its safety and effectiveness, minimal invasiveness, and the ability to preserve organ without damage to endocrine function.^1–3^ In clinical practice, HIFU dosage directly determines the effect of treatment, especially for ultrasound-guided HIFU (USgHIFU) ablation, in which, because of the technically impossible intraoperative temperature measurement, the main concern is the relationship between the dose to be delivered and the type of the fibroids.

The coagulative necrosis area formed in biological tissues through a single focused ultrasound sonication is defined as a biological focal region (BFR), which is the basis for HIFU ablation of biological tissues.^4^ BFR corresponds to the physical focal region of HIFU, namely, acoustic focal region, whose size and form are mainly decided by the physical parameters of transducer such as frequency, ultrasound intensity, and sonication time. For invariable parameters, the human tissue would play a determining role in the dose–effect relation of HIFU treatment. The amount of energy required for tissue ablation per unit volume, defined as energy efficiency factor (EEF), is the quantitative index of dose delivery. The factors influencing EEF include the nature and thickness of tissues in the acoustic pathway, and structure, density, blood supply, and function of target tissues.^5,6^

Previous studies mainly focused on assessing the energy required and the effect of HIFU ablation on the basis of the magnetic resonance imaging (MRI) signals of uterine fibroids.^7,8^ These studies all found that for the treatment of myoma with high T2-weighted signal intensity and obvious enhancement on T1-weighted images, higher energy would be consumed, and the fractional ablation was often unsatisfactory, with more frequent and obvious adverse reactions. In clinical practice, we found that besides the MRI signal intensity, the tissues in the acoustic pathway also greatly influence dose delivery and treatment effect. Therefore, this article, through a retrospective study of a large scale of samples, evaluated the relationship between ablation effect and EEF, determined the factors affecting EEF through multifactor analysis, and explored the dosimetric characteristics of HIFU for uterine fibroids, so as to establish a dosimetry model based on EEF, in the hope of optimizing the clinical protocols and improving the safety and effectiveness of HIFU for uterine fibroids.

## MATERIALS AND METHODS

### Subjects

The protocol was approved by the Ethics Committee of Chongqing Medical University, Chongqing, China. Four hundred and three premenopausal women with symptomatic uterine fibroid who underwent USgHIFU treatment at the First Affiliated Hospital of Chongqing Medical University from February 2012 to February 2014 were retrospectively analyzed.

Inclusion criteria were as follows: the patients’ age was >18 years and premenopausal; all patients had symptoms; the acoustic pathway was safe and the ultrasonic imaging of fibroids was clear; patients could smoothly communicate with the nurse or physician during the procedure; and all patients agreed to undergo pretreatment and posttreatment plain with enhanced MRI scan. Exclusion criteria were as follows: pregnant women; patients with contraindications for MRI scan or gadolinium-injection solution; patients suspected or confirmed of malignant uterine tumor; patients with acute pelvic inflammatory disease or uncontrolled systemic disease; patients unable to lie in a prone position for 2 hours; patients with a history of serious collagenic connective tissue diseases or receiving high dose of radiotherapy for malignant tumors in lower abdomen; and patients with scar in acoustic pathway, causing obvious attenuation of B-model ultrasound in detecting tissues behind, and width of scar being over 1 cm.

### USgHIFU Therapeutic System

The treatment was performed with a US-guided HIFU tumor therapeutic system (Model-JC Focused Ultrasound Tumor Therapeutic System, Chongqing Haifu Medical Technology Co., Ltd., Chongqing, China), combined with B-mode ultrasound (Esaote MyLab70, Genoa, Italy) to provide a real-time imaging during treatment. The transducer with a diameter of 20 cm worked at a frequency of 0.8 MHz. The focal length was 18 cm, with a focal region of 1.5 × 1.5 × 10 mm. The integrated transducer controlled by a computer was fixed in a sac filled with degassed water and could be moved smoothly in 6 directions. During the treatment, an adjustable degassed water balloon was placed between the transducer and the skin anterior to the treated area to push away the bowel in the acoustic pathway.

### Pretreatment MRI

All patients underwent pretreatment MRI with a standardized protocol. T1-weighted imaging (T1WI), T2-weighted imaging (T2WI), and enhanced T1WI were performed with a 1.5T MR unit (Signa Excite-II; General Electric Company, Fairfield, Connecticut). All MRI images were evaluated and measured by 3 experienced radiologists. The targeted fibroids were measured in 3 dimensions: longitudinal (D1), anteroposterior (D2), and transverse (D3). Fibroid volume and nonperfused volume (NPV) were calculated according to the following equation: V = 0.5233 × D1 × D2 × D3.^9^

**FIGURE 1:**
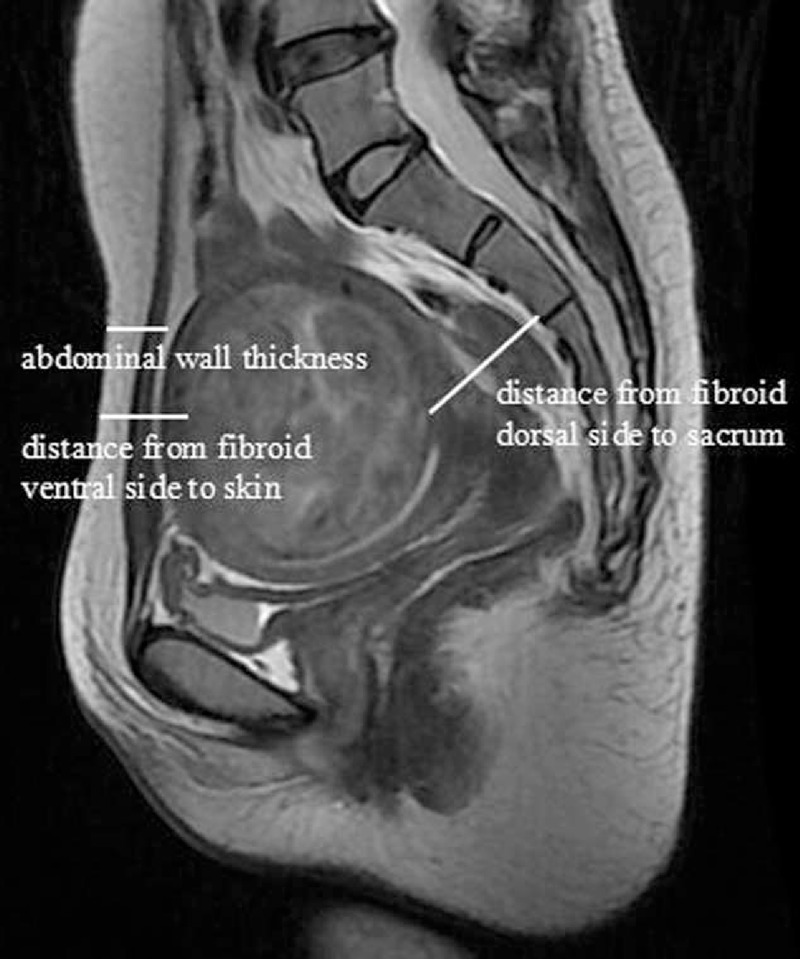
Measurement of distance from uterine fibroid ventral side to skin, uterine fibroid dorsal side to sacrum, and abdominal wall thickness.

Data were recorded as follows: locations of uterine fibroids: anterior wall, lateral wall, posterior wall, and with uterine fibroids in fundus recorded as those in the anterior wall; types of uterine fibroids: intramural, submucous, and subserous; uterus locations: anteposition and retroposition; size of fibroids: the maximal diameter in 3 dimensions; distance from fibroid ventral side to skin: the shortest distance from ventral side to skin in T2WI sagittal image of maximal diameter of fibroid (measurement as shown in Fig. 1); distance from fibroid dorsal side to sacrum: the shortest distance from dorsal side to sacrum in T2WI sagittal image of maximal diameter of fibroid (measurement as shown in Fig. 1); abdominal wall thickness: the thickness of skin, subcutaneous fat, rectus abdominis, and peritoneum on inferior border of the second sacral vertebrae according to T2WI sagittal image (measurement as shown in Fig. 1); signal intensity on T2WI; and enhancement type on T1WI.

**FIGURE 2:**
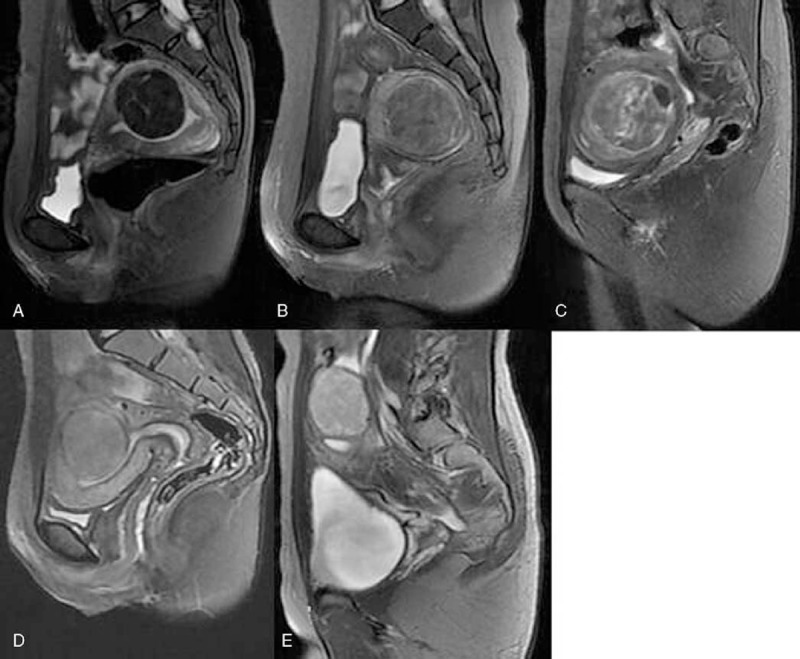
T2-weighted sagittal magnetic resonance imaging before treatment: (A) hypointense; (B) isointense; (C)–(E) hyperintense, in which (C) is heterogeneous and (D) and (E) are homogeneous; (D) slightly homogeneous hyperintense fibroid; and (E) markedly homogeneous hyperintense fibroid.

Uterine fibroids were conventionally classified into 3 types according to pretreatment T2-weighted MRI^10^—hypointense: signal intensity equal to that of skeletal muscle; isointense: signal intensity lower than that of myometrium but higher than that of skeletal muscle; and hyperintense: signal intensity equal to or higher than that of myometrium. The hyperintense fibroids were further classified into 3 groups by Zhao et al^8^: heterogeneous hyperintense fibroids: fibroids with bar (>5 mm) or lamellar, and with high signal intensity that approximates to that of endometrium, or bar (>5 mm) or lamellar, and with low signal intensity that approximates to that of skeletal muscle; markedly homogenous hyperintense fibroids: fibroids with uniformly distributed high signal intensity that was markedly higher than that of myometrium and approximates or equals to endometrium; and slightly homogenous hyperintense fibroids: fibroids with uniformly distributed high signal intensity equal to or slightly higher than that of myometrium (Fig. 2). Accordingly, uterine fibroids in this study were classified into 5 categories: hypointense, isointense, heterogeneous hyperintense, markedly homogenous hyperintense, and slightly homogenous hyperintense.

**FIGURE 3:**
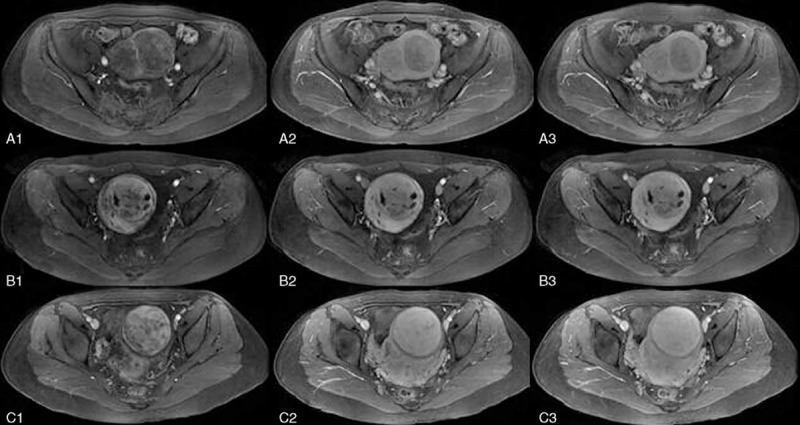
Dynamic contrast-enhanced axial T1-weight magnetic resonance imaging before treatment: A1–A3, B1–B3, and C1–C3 are images acquired at 20, 40, and 60 seconds after contrast injection (from left to right), respectively. (A) Fibroid shows slight enhancement; (B) fibroid shows irregular enhancement; and (C) fibroid shows progressive enhancement.

The arterial vessels within tumors appeared 10 to 30 seconds after intravenous injection of gadolinium, immediately following the perfusion of myometrial vessels, and disappeared 60 seconds after injection.^11^ So, according to the degree of enhancement of uterine fibroids compared to myometrium within 60 seconds after gadolinium injection, the enhanced TIWI were divided into light enhancement, irregular enhancement, and progressive enhancement. The classification was detailed as follows: slight enhancement: the enhancement degree of uterine fibroid was lower than that of myometrium, either homogeneous or heterogeneous; progressive enhancement: the distribution of signal enhancement was homogeneous, the signal enhancement degree equal to or higher than that of the myometrium of uterus; and irregular enhancement: distribution of signal enhancement was heterogeneous, with alternate distribution of enhanced signal or slightly enhanced signal or spotted and lamellar nonenhanced signal. (Fig. 3)

### Pretreatment Preparation

Specific bowel preparations were required for the patients 3 days before treatment, including semiliquid diet and then liquid diet, 2 times of catharsis within 12 hours before treatment, and an enema in the morning of the treatment day. In skin preparation, any scar in acoustic pathway was recorded, and the lower abdominal wall was shaved from the umbilicus to the level of upper margin of the pubic symphysis, and to axillary line on both sides, with a range equal to that in hypogastric operation, leaving no hair or damage on the skin. The patient was trained to increase the filling of bladder to help push away the bowel in the acoustic pathway, and a urinary catheter was inserted to control the bladder volume during treatment.

### Sedation and Analgesia

HIFU ablation procedure was performed under intravenous conscious sedation (fentanyl, 0.8–1 μg/kg, repeated administration at an interval of 30–40 minutes; midazolam hydrochloride, 0.02–0.03 mg/kg, repeated administration at an interval of 30–40 minutes) to a degree of 2 to 3 according to Ramsey Grading. This was to help relieve discomfort and prevent voluntary or involuntary movement of the patients. All patients always remained conscious and the vital signs such as respiration, oxygen saturation, heart rate, and blood pressure were monitored during treatment.

### USgHIFU Ablation

The procedure was performed by licensed doctors in HIFU. The patients were placed in prone position on the HIFU table, with the anterior abdominal wall in contact with a degassed water balloon. The target area and adjacent structure was observed with dynamic real-time ultrasonographic imaging. During the procedure, the therapeutic energy was adjusted based on the feedback from the patient and changes in gray scale on ultrasonographic imaging. This process was repeated on a section-by-section basis to achieve complete ablation of the planned treatment fibroid volume; once the gray scale covered most of the lesion, the treatment was terminated. After treatment, every patient lay in prone position for 2 hours in the observation room before leaving the hospital or returning to ordinary ward. Oral prophylactic antibiotics were suggested for 3 days after treatment. All patients were required to take contraceptive measures within 6 months after treatment.

Observation indexes included overall treatment time (min, defined as the time from the first sonication to the last sonication); sonication power (W, average power of transducer during sonication); sonication time (second, the total time of sonication); treatment intensity (s/h, the sonication time per hour in treatment); treatment efficiency (mm^3^/s, ablated fibroid volume per second in sonication); and EEF (J/mm^3^, the energy required to ablate per unit volume of the fibroid).

### Posttreatment MRI

Plain and enhanced MRI scan were performed 1 month after treatment to assess the ablation effect. The fibroid size was measured with T2WI; NPV, usually considered as fibroid necrosis volume, was measured with enhanced images. The fractional ablation was defined as NPV/fibroid volume × 100%.

### Dosimetric Analysis Method

Dosage is expressed by EEF^5,12^: EEF = η×P × t/V (J/mm^3^), where η indicates focusing factor (=0.7), P indicates sonication power (W), t indicates sonication time, V indicates NPV (mm^3^), and EEF indicates the energy required to ablate per unit volume of the fibroid.

### Statistical Analysis

Normally distributed data are reported as the mean ± standard deviation; skewly distributed data are reported as median and interquartile range. A multifactor linear regression model was established with stepwise regression method. The data was analyzed with SPSS software (SPSS 19.0, IBM Company, Chicago, IL) and *P* value <0.05 was considered statistically significant.

## RESULTS

### Patients and Fibroids

A total of 403 patients were treated by USgHIFU. In multiple fibroids cases, only the first treated fibroid was recorded, because in the treatment of the first fibroid, ultrasonic energy in the same acoustic pathway would be absorbed by the other fibroids, which would affect the sonication time and treatment dosage of the nonfirst treated fibroids. The patients’ mean age was 37.4 ± 6.5 years (range, 21–52 years); the mean body mass index (BMI) was 21.7 ± 2.7 kg/cm^2^ (range, 15.6–30.5 kg/cm^2^); the mean size of fibroid was 58.1 ± 18.4 mm (range, 24.8–140.2 mm); the mean abdominal wall thickness was 31.5 ± 8.0 mm (range, 14.8–70.2 mm); the mean distance from fibroid dorsal side to sacrum was 23.5 ± 15.7 mm (range, 1.8–86.1 mm); and the mean distance from fibroid ventral side to skin was 49.8 ± 22.9 mm (range, 11.2–110.4 mm) (Table 1).

**TABLE 1:**
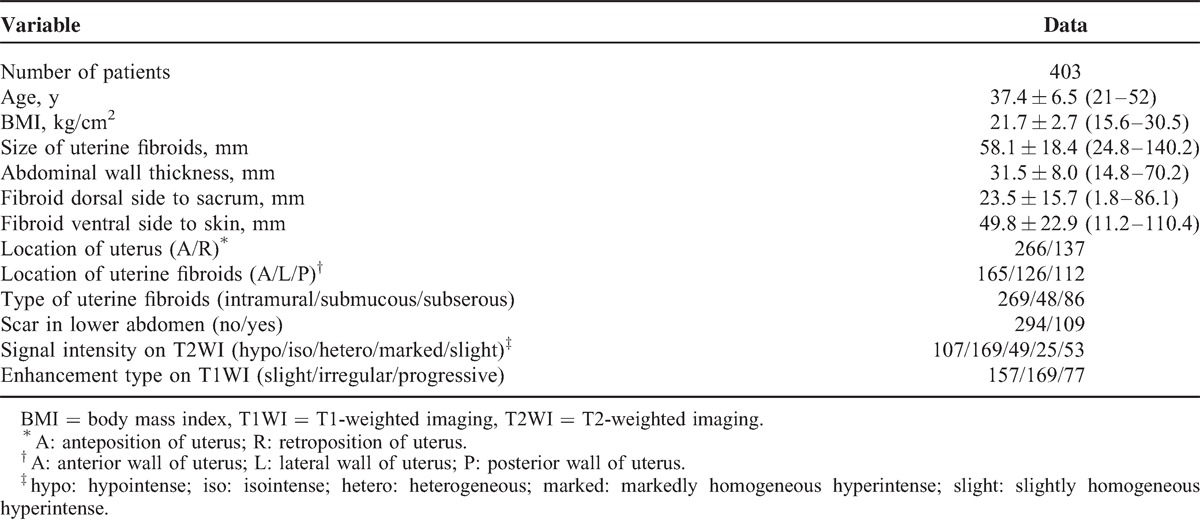
Baseline Data of Patients With Uterine Fibroids

### Postprocedure Evaluation

The mean acoustic sonication power was 400 ± 22 W (range, 219–499 W); the median treatment time was 92.0 minutes (interquartile range, 60.0–140.0 minutes); the median sonication time was 1141 seconds (interquartile range, 688–1937 seconds); the mean fractional ablation was 77.5 ± 19.1% (range, 6.1%–100.0%); and the median EEF was 6.3 J/mm^3^ (interquartile range, 3.3–14.0 J/mm^3^) (Table 2).

**TABLE 2:**
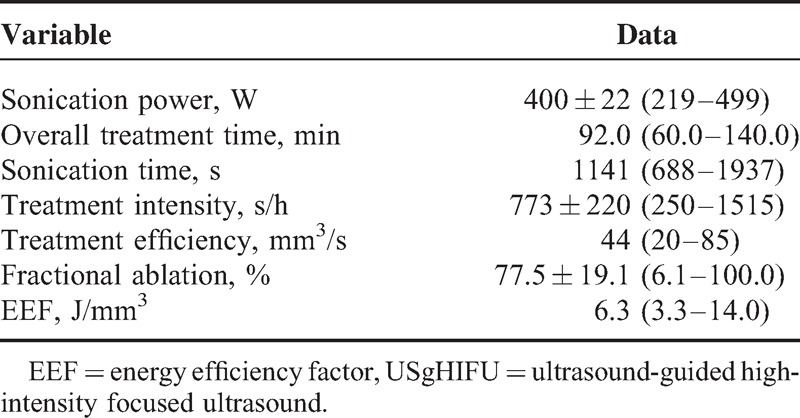
Treatment Results of Uterine Fibroids Treated by USgHIFU

### Establishment of Dosimetric Model

The EEF was set as dependent variable, and a total of 12 factors were established as independent variables including age, BMI, location of uterus, location of uterine fibroids, type of uterine fibroids, size of uterine fibroid, abdominal wall thickness, distance from fibroid dorsal side to sacrum, distance from fibroid ventral side to skin, signal intensity on T2WI, enhancement type on T1WI, and scar in lower abdominal (yes or no). The correlation between 12 predictors and EEF is shown in Table 3. Multiple linear regression analysis was conducted with a stepwise regression model. There were 7 statistically significant predictors: size of uterine fibroid (negative correlation), distance from fibroid ventral side to skin (positive correlation), location of uterus (positive correlation), location of uterine fibroids (positive correlation), type of uterine fibroids (negative correlation), signal intensity on T2WI (positive correlation), and enhancement type on T1WI (positive correlation). The 7 predictors were included one by one into the linear regression model when *P* value was ≤0.05, and were eliminated when *P* value was ≥0.1. The results showed that distance from fibroid ventral side to skin, enhancement type on T1WI, size of uterine fibroid, signal intensity on T2WI were included into linear regression model one by one. The predictor that was first included had the strongest correlation and also the closest relationship with the model. The predictors in the model were all statistically significant (*P* < 0.05), whereas other predictors had no obvious correlation with EEF (*P* > 0.05). The regression model is shown in Table 4.

**TABLE 3:**
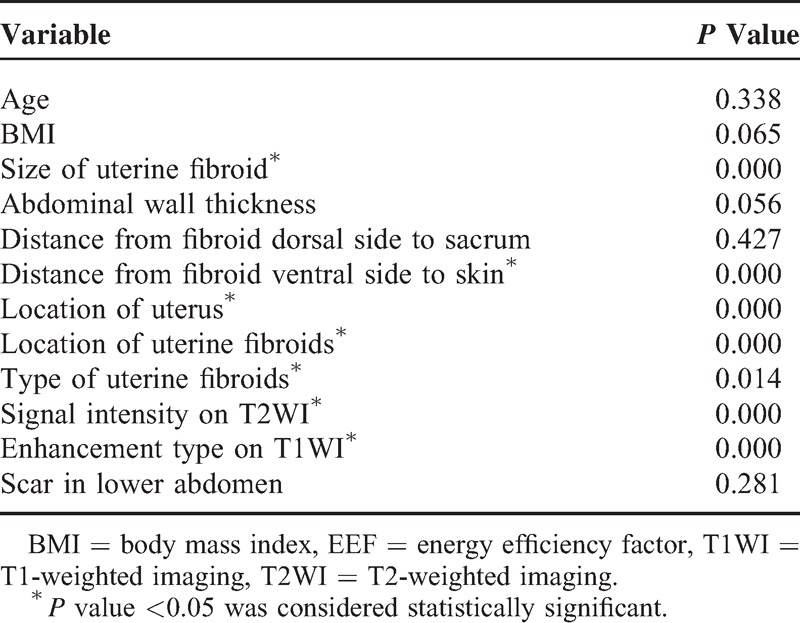
Correlation Between Predictors and EEF

**TABLE 4:**
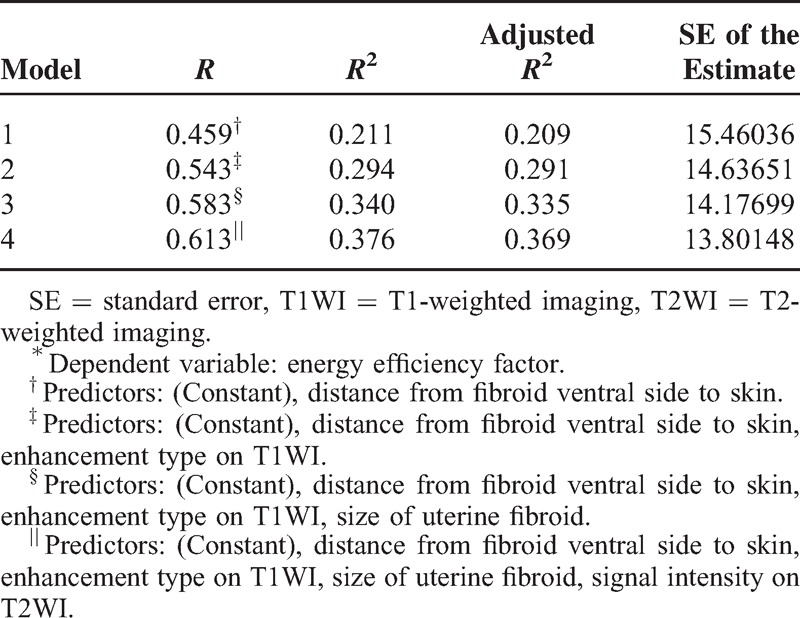
Multivariable Regression Model^∗^

**FIGURE 4:**
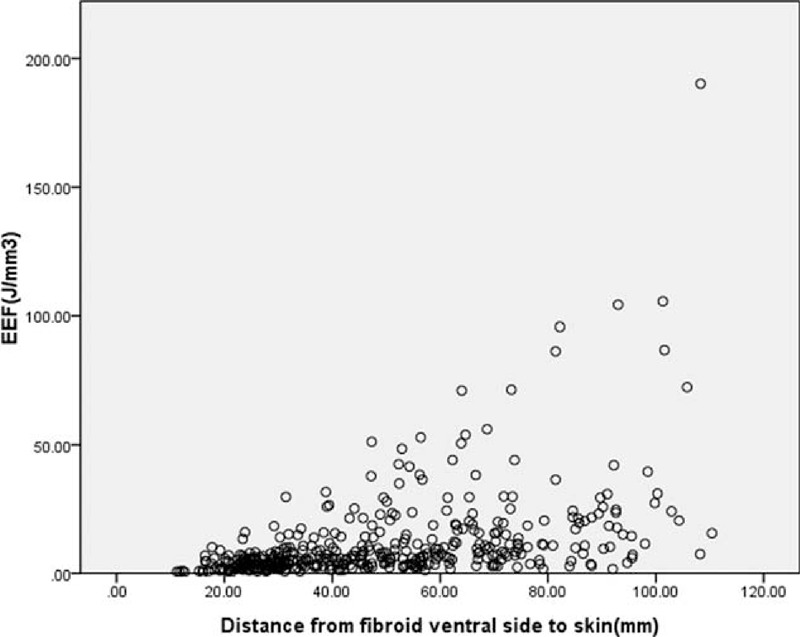
Scatterplot of energy efficiency factor distribution correlated with distance from fibroid ventral side to skin.

**FIGURE 5:**
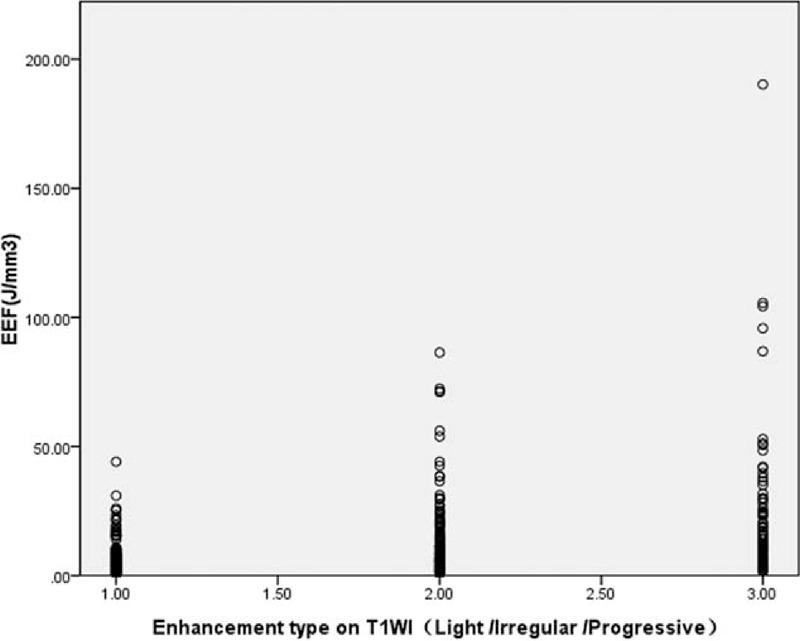
Scatterplot of energy efficiency factor distribution correlated with enhancement type on T1-weighted imaging.

**FIGURE 6:**
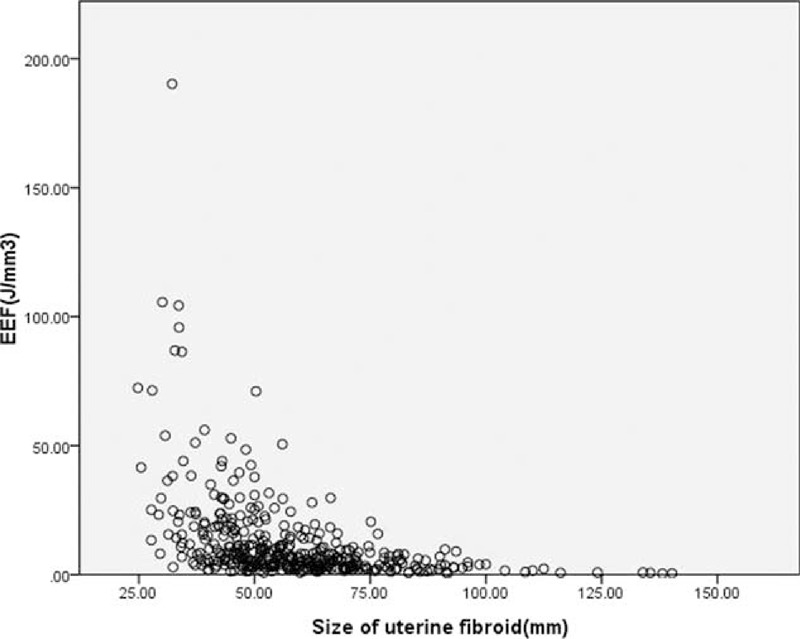
Scatterplot of energy efficiency factor distribution correlated with size of uterine fibroid.

**FIGURE 7:**
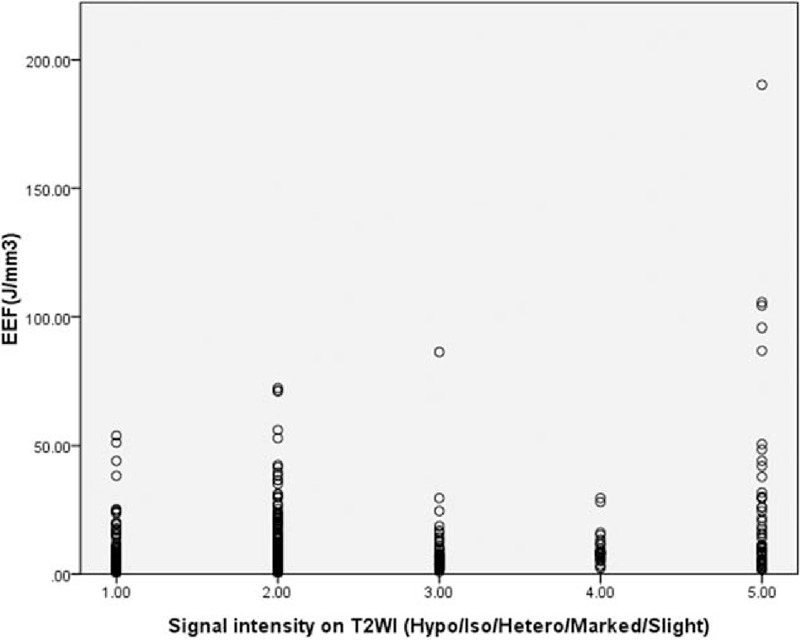
Scatterplot of energy efficiency factor distribution correlated with signal intensity on T2-weighted imaging.

Four multiple regression models were established: Models 1–4, but the goodness of fit of Model 4 was better than that of Models 1–3 (*R*^2^, 0.376 > 0.340 > 0.294 > 0.211). Through analysis of variance (Table 5), the probability value of *F* statistic was 0.000 in Model 4. Since 0.000 < 0.01, and with the introduction of predictors, its significance probability value was far <0.01, therefore, null hypothesis that overall regression coefficient was 0 could be significantly rejected. At the same time, by the analysis of variance, a linear relationship was found between the dependent variable EEF and predictors (distance from fibroid ventral side to skin, enhancement type on T1WI, size of uterine fibroid, and signal intensity on T2WI) (Figs. 4–7).

**TABLE 5:**
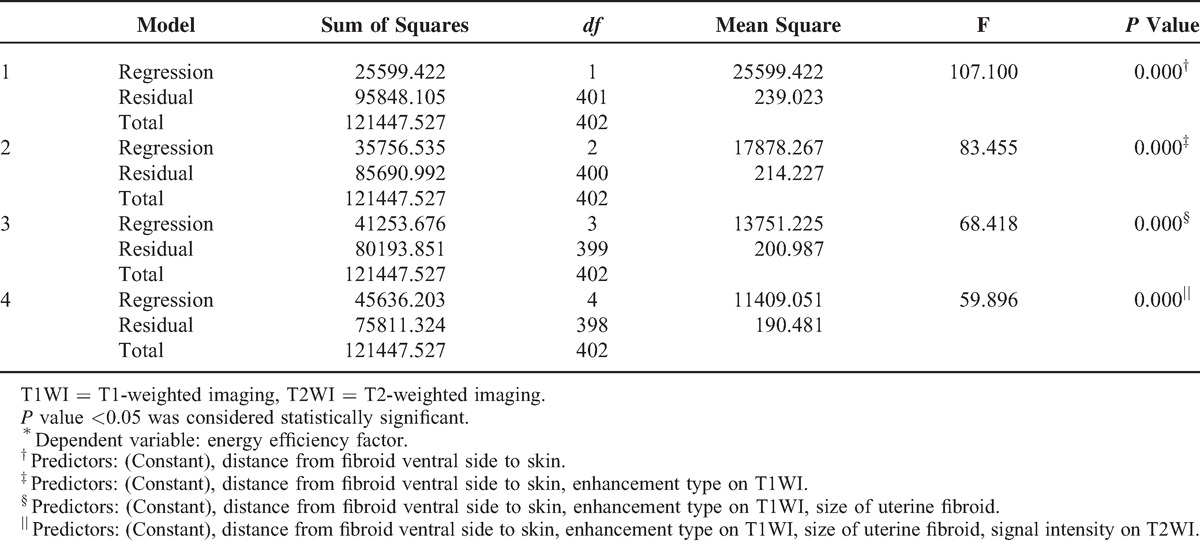
Analysis of Variance^∗^

In Table 6, the Sig of constant was 0.563 > 0.05 in Model 4, which was not significant. Since the “constant” in the standard coefficient had no numerical value, it was removed. Therefore, the multiple linear regression equation should be ý = 0.233 × X1 + 5.623 × X2 – 0.235 × X3 + 2.648 × X4, where variable ý = EEF; X1 = distance from fibroid ventral side to skin; X2 = enhancement type on T1WI (slight enhancement = 1, irregular enhancement = 2, progressive enhancement = 3); X3 = size of uterine fibroid; and X4 = signal intensity on T2WI (hypointense = 1, isointense = 2, heterogeneous hyperintense = 3, markedly homogenous hyperintense = 4, slightly homogenous hyperintense = 5). The collinearity statistics in Table 6 shows that tolerance of each predictor was >0.1 and variance inflation factors were all <10. So, no collinearity was found in the 4 predictors. If the predictors were random predictors, multiple regression could not be explained by the assumption that there was collinearity among predictors. In this study, the “characteristic value” method, which was used to describe the variance of predictor and detect the presence of strong multicollinearity among predictors, was adopted for “collinearity diagnosis.” As Table 7 shows, single characteristic value could not explain “distance from fibroid ventral side to skin,” “enhancement type on T1WI,” “size of uterine fibroid,” and “signal intensity on T2WI” at the same time. So, there was no collinearity among the 4 predictors, and the model matched well with the requirements of the statistics.

**TABLE 6:**
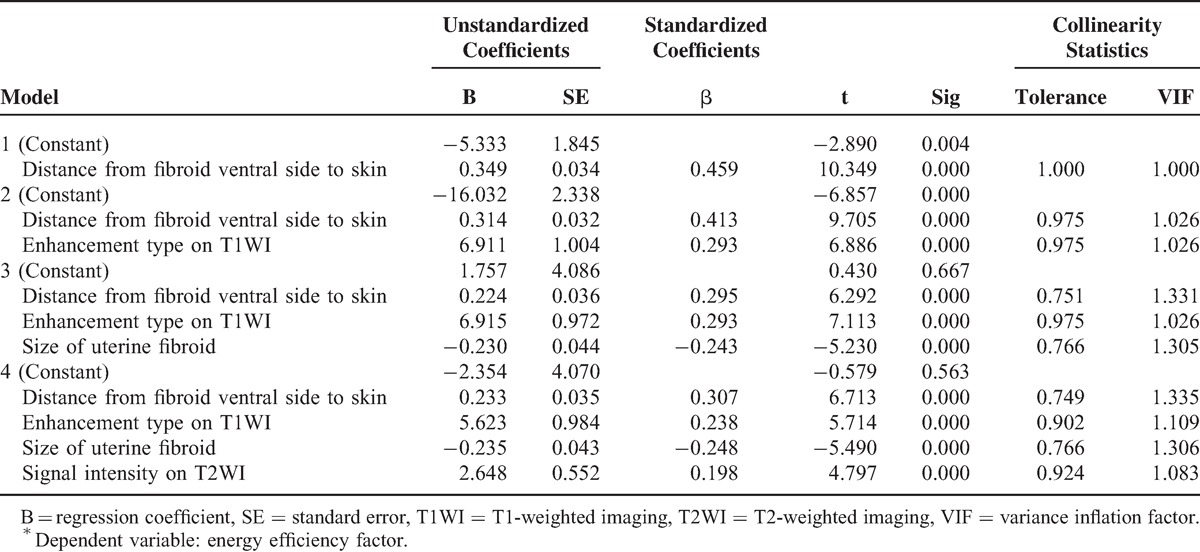
Coefficient of Multivariable Regression Model^∗^

**TABLE 7:**
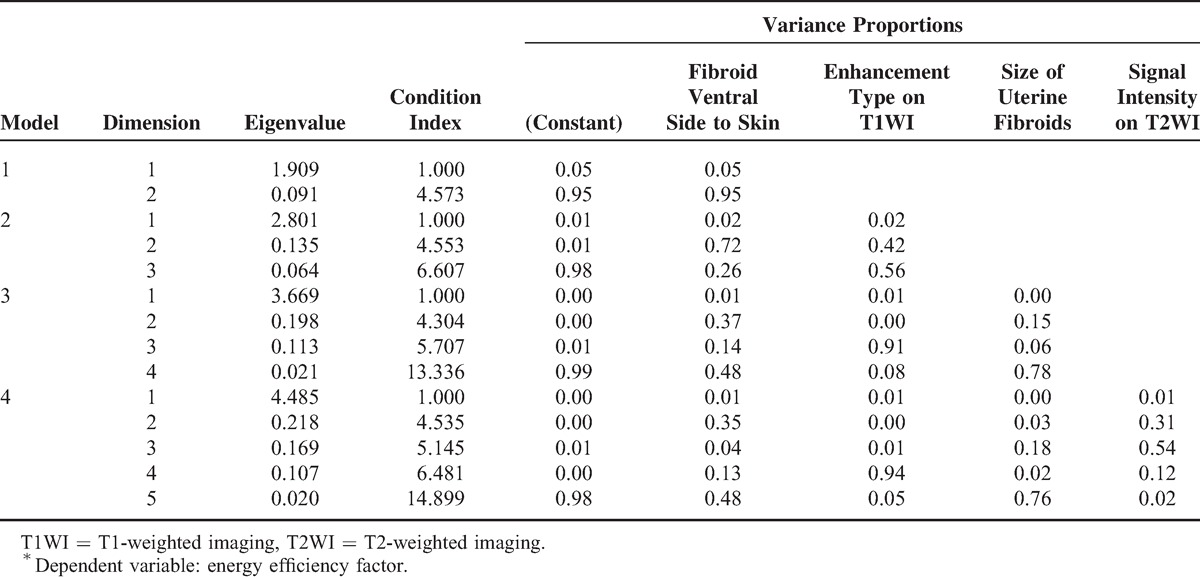
Collinearity Diagnosis^∗^

## DISCUSSION

In this study, 7 predictors had linear correlation with dependent variable EEF: size of uterine fibroid, distance from fibroid ventral side to skin, location of uterus, location of uterine fibroids, type of uterine fibroids, signal intensity on T2WI, and enhancement type on T1WI. With the introduction of multiple predictors into a regression model, the statistical significance of the other predictors in the model was tested when each new predictor was added. If there were predictors showing no statistical significance, then the predictors that contributed minimally to dependent variable and showed no statistical significance would be removed one by one from the regression model. In turn, the predictors were included or excluded until they all showed statistical significance in the regression model, and the introduction of any predictors into the regression model was not statistically significant any more. Because of the insignificant contribution of “location of uterus,” “location of uterine fibroids,” and “type of uterine fibroids” to the EEF, they were removed from the regression model. Individually, these 3 predictors had effects on delivering energy: retroposition of uterus were more difficult for ablation than anteposition of uterus; fibroids in posterior wall of uterus were more difficult for ablation than anterior and lateral wall of uterus; and intramural fibroids needed more energy than submucous and subserous fibroids. But they are not the main factors, especially the “location of uterus” and “location of uterine fibroids” affecting EEF belong to the category of the distance of acoustic pathway. However, the “distance from fibroid ventral side to skin” reflects more accurately the distance of acoustic pathway. Therefore, 4 predictors were finally incorporated into the regression model: “distance from fibroid ventral side to skin,” “enhancement type on T1WI,” “size of uterine fibroid,” and “signal intensity on T2WI.”

Tissues in the acoustic pathway will absorb, reflect, and scatter ultrasonic waves when focused ultrasound is applied in ablation of deep target, and consequently result in ultrasonic energy attenuation with an increasing amount of tissues in front of the focus. Studies have shown that the deeper the tissue was, the more the energy was required to ablate the same volume of tissue in vitro with equal damage effect.^13^ Li et al^5^ studied the relationship between EEF and focus depth and discovered that the ultrasonic energy required to ablate the same unit volume of target tissue had positive correlation with focus depth. In this study, the regression model with EEF as the dependent variable showed that the EEF and distance from fibroid ventral side to skin (the thickness of tissues in acoustic pathway except target leiomyoma, such as skin, subcutaneous fat, muscle, peritoneum, bladder, and myometrium) was positively correlated. The longer the distance from fibroid ventral side to skin was, the more ultrasonic energy was needed to ablate the fibroid. Thus, we further verified the theory applied in the HIFU ablation of uterine fibroids. However, no significant correlation between abdominal wall thickness and the EEF was found in this study, which might be caused by the following reasons: the distribution range of abdominal wall thickness was narrow, with the mean thickness of 31.5 ± 8.0 mm (range, 14.8–70.2 mm) and the thickness of <4 cm in 354 patients (87.8%), which suggested no significant correlation between this range of abdominal wall thickness and the EEF; during the procedure, lower abdominal wall was pushed out of the acoustic pathway by water balloon and thus was greatly compressed, which further reduced the attenuation effects on ultrasonic energy; and one of the inclusion criteria for this retrospective study was that through simulation, acoustic pathway should be clear and safe, and myoma should be clearly visible by monitoring ultrasound before treatment, which means if the abdominal wall was too thick to influence the display of myoma by monitoring ultrasound, this portion of patients would be excluded from the study. In addition, the scar in lower abdominal wall in the acoustic pathway should greatly affect the energy absorption and attenuation. But the results of this study showed that whether there was lower abdominal scar in the patients had no obvious correlation with EEF. The main reason could be that the patients with lower abdominal scar in acoustic pathway that caused the tissues behind to be invisible by B-model ultrasound or with scar of >1 cm in width met the exclusion criteria of this study. So, after exclusion of these patients, no obvious effect on acoustic energy was found in the other patients with scar.

There was a direct relationship between enhanced degree of fibroid on dynamic enhancement MRI and artery blood supply of fibroid, and progressive enhancement indicated that arterial blood supply to the fibroid was rich. This study showed that the dosage for ablating the progressive enhancement fibroids was significantly higher than that for the slight enhancement and irregular enhancement ones, and the type of enhancement degree was positively correlated with the EEF. The main reason is that energy deposition in tissue is influenced by the cooling effect of blood flow that can quickly take away ultrasonic heat and decrease the local temperature,^14,15^ and ultrasound energy is not easy to be deposited in progressive enhancement fibroids because of more cells, less collagen fiber, and low density in the fibroids.^16^ In contrast, the blood supply of the slight enhancement fibroids is not so rich, and the irregular enhancement fibroids have ischemic region; as a result, these fibroids are easy to be ablated because of easy deposition and diffusion of ultrasound energy. So, single HIFU ablation of the progressive enhancement fibroids needs more energy, and a combined HIFU ablation for this category of fibroids is recommended. Li et al^17^ have confirmed that a combination with pretreatment injection of iodized oil can significantly improve the efficiency of HIFU ablation.

T2WI signal type of fibroid had linear correlation with EEF, primarily because different T2WI signal fibroids have different histological structures.^8,18,19^ Based on previous studies, the pathologic structure of slightly homogeneous hyperintense fibroids was dominant in cell component, with less collagen fiber and low myoma density; the absorption coefficient of ultrasonic energy was low, and the fibroids often had rich blood supply, affecting the absorption of ultrasonic energy. Thus, the ablation is difficult. Internal degenerations such as cystic degeneration, mucoid degeneration, and reddish degeneration, as well as poor blood supply, make it easier to ablate heterogeneous hyperintense fibroids and the markedly homogeneous hyperintense fibroids. The hypointense and isointense fibroids, especially the hypointense fibroids, lacking liquid and mucin between cells, are typically composed of closely arranged cells and high proportion of collagen fibers, so these fibroids are likely to absorb and deposit ultrasonic energy. Therefore, for slightly homogeneous hyperintense fibroids,^2^ if dynamic contrast-enhanced MRI shows progressive enhancement, and the fibroid is not big, with a lot of tissues in the acoustic pathway, single HIFU for these fibroids is not recommended; other treatments or combined therapies, such as HIFU combined with gonadotropin-releasing hormone agonist, according to some studies, could improve the effectiveness of HIFU treatment.^10^

This study showed that the size of uterine fibroid and EEF were negatively correlated, which means that the larger the size of uterine fibroid was, the less dosage was required to ablate the same volume of fibroid. The possible reasons for this result would be as follows: first, the uterine fibroids of larger size occupy larger area of acoustic pathway area, absorbing more energy, and the ultrasonic environment of fibroid tissue outside of the focus is dynamically changed along with the range of necrosis expansion and temperature increasing at the focus, thereby facilitating the deposition of ultrasound energy. Experimental study has confirmed that the EEF of surface damage is less than that of line damage, and the EEF of volumetric damage is less than that of surface damage,^5^ which can be used as theoretical basis for this phenomenon. Second, circulatory disorder often exists inside large fibroids so that the structure of large fibroids usually changes with degenerations such as glassy degeneration, causing an increase of density thus raising the absorption coefficient of ultrasonic energy. Besides, the energy delivered is not easily lost with the blood flow. Therefore, it is relatively easy to ablate large uterine fibroids with high efficiency.

The limitations of this retrospective study are 2-fold. First, the ablation procedures performed by different doctors could have influence on dosage delivery. Besides, in the measurement of the distance from fibroid ventral side to skin distance by MRI, patients were in supine position, but during the treatment, the patients were in prone position, which could have changed the distance a little. So, we used a degassed water balloon that was placed between the transducer and the anterior abdominal wall to push away the bowel from the acoustic pathway to minimize the deviation of measurement. Future studies should be more standardized to minimize any possible interference.

In conclusion, EEF, as quantitative index, directly reflects the relationship between dosage and the efficiency of HIFU. Besides blood supply status of fibroids (enhancement type on T1WI) and tissue structure of fibroids (signal intensity on T2WI) influencing the relationship between dosage and effectiveness of HIFU, the distance from fibroid ventral side to skin (tissue thickness in acoustic pathway) and size of uterine fibroid are also important factors for dosage delivery. During HIFU for uterine fibroids, “distance from fibroid ventral side to skin,” “enhancement type on T1WI,” “size of uterine fibroid,” and “signal intensity on T2WI” can be used as predictors to guide clinical dosage delivery, which are readily available in clinic. At the same time, the dosimetry model that can predict dosage delivery of HIFU for uterine fibroid has been established in this study: ý = 0.233 × X1 + 5.623 × X2 – 0.235 × X3 + 2.648 × X4, where variable ý = EEF; X1 = distance from fibroid ventral side to skin; X2 = enhancement type on T1WI (slight enhancement = 1, irregular enhancement = 2, progressive enhancement = 3); X3 = size of uterine fibroid; and X4 = signal intensity on T2WI (hypointense = 1, isointense = 2, heterogeneous hyperintense = 3, markedly homogenous hyperintense = 4, slightly homogenous hyperintense = 5). The results of this study provide a method for the prognosis of HIFU for uterine fibroids and optimization of indications.
